# The β‐Chain Mutation p.Arg17Stop Impairs Fibrinogen Synthesis and Secretion: A Nonsense Mutation Associated With Hypofibrinogenemia

**DOI:** 10.1002/jcla.25123

**Published:** 2024-12-12

**Authors:** Chen Qian, Chaoyu Huang, Qinghui Luo, Kaili Qin, Yangyang Wu, Lin Liao, Qian Zhang, Liqun Xiang, Jie Yan

**Affiliations:** ^1^ Department of Clinical Laboratory The First Affiliated Hospital of Guangxi Medical University, Key Laboratory of Clinical Laboratory Medicine of Guangxi Department of Education Nanning, Guangxi Zhuang Autonomous Region People's Republic of China; ^2^ Organizing Personnel Section The First Affiliated Hospital of Guangxi Medical University Nanning, Guangxi Zhuang Autonomous Region People's Republic of China

**Keywords:** *FGB* mutation, hypofibrinogenemia, nonsense mutation

## Abstract

**Background:**

Congenital hypofibrinogenemia, a quantitative fibrinogen disorder, is characterized by abnormally low levels of both functional and antigen fibrinogen. We identified a heterozygous nonsense mutation, p.Arg17Stop, in the fibrinogen Bβ chain of a three‐month‐old female infant.

**Methods:**

Coagulation testing, gene analysis, in vitro plasmid construction, and functional analyses were conducted to investigate the underlying pathogenic mechanisms.

**Results:**

Plasma fibrinogen levels showed decrease in the proband. DNA sequencing of the proband revealed a heterozygous point mutation (c.139C>T) in exon 2 of the FGB gene causing Arg → Stop substitution. Human Arg17 was found to be highly conserved. In vitro expression analyses indicated that the mutation impacts both the transcription and translation of the FGB gene, subsequently affecting the synthesis and secretion of fibrinogen.

**Conclusions:**

The p.Arg17Stop mutation in the fibrinogen Bβ chain disrupts fibrinogen production and secretion, contributing to hypofibrinogenemia.

## Graphical abstract

We reported the first case of a FGB p.Arg17Stop mutation with a heterozygous gene status, and conducted research on the pathogenic mechanisms related to the fibrinogen Bβ (p.Arg17Stop) mutation. This mutation significantly impairs both the synthesis and secretion of fibrinogen.

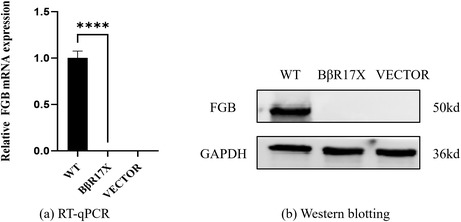

## 1 Introduction

Fibrinogen is a vital protein synthesized and secreted by hepatocytes, playing a key role in the coagulation and hemostasis processes of the human body [1]. Fibrinogen has a molecular weight of 340 kDa, composed of two identical sets of three polypeptide chains: Aα, Bβ, and γ [2]. The Bβ‐chain serves as the rate‐limiting factor in the hepatic production of the fibrinogen hexamer [3]. The *FGA* gene encodes the Aα‐chain and is located on 4q31.3 [4]. The *FGB* gene encodes the Bβ‐chain, which is located on the same chromosome (4q31) as the *FGA* gene but oriented antisense to it [1]. The *FGG* gene, encoding the γ‐chain, is located on chromosome 4q32.1 [5]. Abnormal synthesis and secretion of fibrinogen often leads to various diseases. Elevated total fibrinogen is generally associated with a higher risk of venous thrombosis, whereas decreased total fibrinogen is associated with a higher risk of the bleeding phenotype. Mutations in any of the three fibrinogen genes can result in congenital fibrinogen disorders (CFD) [6]. CFD includes two subtypes: qualitative and quantitative fibrinogen disorders [7]. Congenital hypofibrinogenemia, a quantitative fibrinogen disorder, is characterized by abnormally low levels of both functional and antigen fibrinogen [8]. The clinical manifestations of congenital hypofibrinogenemia are heterogeneous, with different genotypes causing varied clinical outcomes [9, 10]. Mutations at the same site may lead to varied clinical manifestations across individuals [6]. Clinical manifestations in patients with hypofibrinogenemia correlate with fibrinogen levels. Typically, hypofibrinogenemia is asymptomatic when fibrinogen levels are above 1 g/L [11]. However, spontaneous bleeding events are common when the plasma fibrinogen concentration drops below 0.5 g/L [11].

In this study, we identified a heterozygous nonsense mutation, p.Arg17Stop, in the fibrinogen Bβ chain of a three‐month‐old female infant. To investigate the pathogenesis of hypofibrinogenemia caused by the p.Arg17Stop mutation in *FGB*, we established a recombinant CHO cell line producing fibrinogen and analyzed the effects of this mutation on fibrinogen assembly and secretion by the cell line.

## 2 Materials and Methods

### 2.1 Patient

The proband is a three‐month‐old female infant. 2 months prior to her presentation, she was found to have a low fibrinogen concentration of 0.65 g/L, though she exhibited no signs of bleeding or other related symptoms. After receiving fibrinogen and plasma infusions at a different hospital, her fibrinogen levels increased to 1.02 g/L. She is now at our hospital for further diagnosis and treatment.

### 2.2 Coagulation Tests

Coagulation tests were performed according to our previous method [12].

### 2.3 DNA Sequence Analysis

The extraction and amplification methods of genomic DNA and sequencing of amplification products were the same as our previous methods [12].

### 2.4 In Vitro Expression

#### 2.4.1 Plasmid Construction

The FGA, FGB, and FGG genes were carefully cloned into the eukaryotic expression plasmid pcDNA3.1(−), resulting in the creation of the wild‐type expression vectors: pcDNA3.1‐Aαwt, pcDNA3.1‐Bβwt, and pcDNA3.1‐γwt. To generate the pcDNA3.1‐BβR17X construct, the pcDNA3.1 wild‐type expression vector served as the template. For the transformation process, competent cells were initially placed on ice. Once thawed, 10 μL of the ligation product was added to the cells, followed by a 30‐min incubation on ice. The cells then underwent a heat shock at 42°C for precisely 90 s, immediately cooled down again on ice for a quick 2–3 min. Post transformation, the cells were incubated in antibiotic‐free SOC medium, with vigorous shaking at 225 rpm, at 37°C for 45 min. The transformed cells were then centrifuged at 3000 rpm for 2 min, after which 900 μL of the supernatant was carefully removed. The remaining pellet was resuspended and plated onto culture media containing either ampicillin or kanamycin, depending on the requirements. These plates were incubated at 37°C overnight, allowing colonies of transformants to emerge. Several colonies were then selected for sequencing and identification to confirm successful transformation, and the validated strains were preserved for further study.

#### 2.4.2 Cell Transfection

1. Cell culture: the Chinese hamster ovary (CHO) cell strain was used in the experiment, and the culture medium was DMEM + 10% FBS + 1%P/S; culturing took place at 37°C under 5% CO_2_. The cells undergo fluid changes every 2 days. Passage was performed when the cell confluence reached 80%.
2. To generate the pcDNA‐Fgwt plasmid, equal parts of pcDNA‐Aαwt, pcDNA‐Bβwt, and pcDNA‐γwt were combined in a precise 1:1:1 ratio. Similarly, to create the pcDNA‐BβR17X plasmid, pcDNA‐Aαwt, pcDNA‐BβR17X, and pcDNA‐γwt were mixed together, also in a 1:1:1 ratio. These meticulously prepared plasmid mixtures, along with the empty pcDNA3.1(−) plasmid, were then introduced into CHO cells through transfection.

Transfection step: Seed the CHO cells into six‐well plates 1 day in advance, and each well adds 3 × 10^5^cells. Transfection was performed the next day when the cell reached 80% confluence. Opti‐MEM medium (125 μL) and Lipofectamine 3000 (5 μL) (mixed well before use) were added to an EP tube and mixed gently to make the Lipofectamine 3000 dilution. Opti‐MEM medium (125 μL) and plasmid DNA (2.5 μg) were added to the EP tube and mixed gently to make a DNA dilution. DNA dilution is added to each tube of diluted Lipofectamine 3000 at a ratio of 1:1 to make the DNA–Lipofectamine 3000 complex. Incubate the DNA–Lipofectamine 3000 complex for 10 min and slowly drip into the inoculated cells.

#### 2.4.3 RT‐qPCR of Wild‐Type, Mutant, and Vector CHO Cells After Transfection

1. Cell total RNA extraction and reverse transcription: after transfection for 48 h, 500 μL of NucleoZOL reagent (MACHEREY‐NAGEL, 740404.200) was added to each well of each 6‐well plate, ensure complete lysis by repeated pipetting. Add 200 μL of RNase‐free water per 500 μL of NucleoZOL to the lysate. Shake the sample vigorously for 15 s. Incubate at room temperature for 5 min. Centrifuge samples for 15 min at 12,000 × *g* at room temperature. Transfer the supernatant to a fresh EP tube. Add 400 μL of isopropanol per 400 μL of supernatant in order to precipitate RNA. Incubate samples at room temperature for 10 min. Centrifuge samples for 10 min at 12,000 × *g*. Remove and discard the supernatant. Use 400 μL of 75% ethanol wash RNA. Centrifuge the pellets for 3 min at 8000 × *g*. Remove ethanol from the pellet by pipetting. Repeat the ethanol washing step. Dissolve the RNA pellet in RNase‐free water to obtain an RNA concentration of 1 μg/μL. Vortex the sample for 3 min at room temperature for efficient solubilization. Reverse transcription was performed according to the instructions of the PrimeScriptTM RT reagent kit with gDNA eraser (Takara, RR047A). The cDNA was stored at −20°C prior to use.
2. Real‐time PCR was performed according to the instructions of the TB Green Premix Ex Taq II (Tli RNaseH Plus) (Takara, RR820A). The reaction system of Real‐time PCR is shown in Table S1. The reaction conditions of Real‐time PCR are shown in Table S2. The primer sequences of FGB and β‐actin are shown in Table S3.
3. Experimental analysis and data statistics.

To ensure accuracy, the dissolution curve was analyzed to rule out any nonspecific amplification. The β‐actin gene served as an internal control, playing a crucial role in normalizing the efficiency of the RT‐qPCR. Data analysis was conducted using the 2 − ΔΔCT method, employing the following specific formula for precise calculations: ΔCT = (CT FGB—CT β‐actin), and ΔΔCT = (ΔCT mutant—ΔCT wild‐type).

#### 2.4.4 Western Blot of Wild‐Type, Mutant, and Vector CHO Cells After Transfection

1. Extraction and denaturation of total cell protein: after transfection for 48 h, the medium was discarded and the cells were washed twice with PBS (Solarbio, P1020) and then cells were scraped into the EP tube. After centrifugation to remove PBS, add 150 μL RIPA buffer (Solarbio, R0010) and 1.5 μL PMSF (Solarbio, P0100) to each EP tube. The cells were disrupted using an ultrasonic cell disruptor at 30% power with an ice bath for 1 minute. The cells are cleaved on ice for 30 min and centrifuged to obtain the supernatant. The protein concentration is measured by the BCA method. Add protein sample loading buffer (Denaturing, Reducing, 5×) (Epizyme, LT101S) to the protein and incubate at 100°C for 10 min.
2. The expression level of fibrinogen Bβ‐chain was detected by western blot.

The protein samples obtained through the above steps were separated by 10% SDS‐PAGE according to molecular weight and then transferred to polyvinylidene fluoride (PVDF) membranes (Merck Millipore). After washing the PVDF membranes once with TBST (Solarbio, T1082), the PVDF membranes were blocked in 5% milk for 1 h at room temperature. PVDF membranes were probed with fibrinogen beta chain polyclonal antibody (Proteintech, 16,747‐1‐AP) and gapdh antibody (Cell Signaling Technology, 2118S) at 4°C overnight and secondary anti‐rabbit antibody (Cell Signaling Technology, 5151S) at room temperature for 1 h. After washing the PVDF membranes three times with TBST, the PVDF membranes were placed in infrared fluorescence scanning film imager for scanning film imaging.

#### 2.4.5 Determination of Fibrinogen Expression in Cell Lysate and Supernatant Post‐Transfection by ELISA

1. Extraction of Cell Lysate and Supernatant: Following the removal of the culture medium, cells were subjected to digestion using pancreatic enzymes, subsequently resuspended in PBS, and disrupted by ultrasonic treatment. The resulting cell lysate was then centrifuged at 1500 × *g* for 10 min, while the cell culture medium underwent centrifugation at 4°C for 20 min at 1000 × *g*. The supernatants from both the lysate and the medium were carefully collected for subsequent analysis.
2. Detection of Fibrinogen Concentrations via ELISA: The supernatants obtained from the previous steps, both from the cell lysate and the culture medium, were loaded into the wells of an ELISA plate. The ELISA procedure was executed according to the detailed protocol provided by the ELISA kit manufacturer (Elabscience, E‐EL‐H6106).

### 2.5 Conservation Analysis and Bioinformatics Analysis

ClustalX2.1 software was used to conduct conservation analysis of amino acid sequences between humans and nine homologous species ( *Bos taurus* , *Canis lupus familiaris* , *Danio rerio* , *Macaca mulatta* , *Mus musculus* , *Pan troglodytes* , *Phasianus colchicus* , *Rattus norvegicus* , *Xenopus laevis* ) using data from the NCBI database (HomoloGene, http://www.ncbi.nlm.nih.gov/homologene).

The Bβ (p.Arg17Stop) mutation was analyzed using the online bioinformatics tool MutationTaster and PROVEAN to assess its potential adverse effects on fibrinogen function.

### 2.6 Molecular Modeling of the Amino Acid Mutations

Retrieve the fibrinogen beta chain amino acid sequence from the NCBI database and construct structural models for both wild‐type and mutant fibrinogen beta chains using the SWISS‐MODEL website (https://swissmodel.expasy.org/). Subsequently, visualize these structures using SWISS‐PDB Viewer software. Obtain 3D structural data for fibrinogen from the Protein Data Bank (PDB) and analyze the impact of the Bβ (p.Arg17Stop) mutation on the spatial structure of the fibrinogen using Swiss‐PDB Viewer software.

## 3 Results

### 3.1 Laboratory Tests

The proband's coagulation profile revealed the following results: prothrombin time (PT) was measured at 12.4 s, activated partial thromboplastin time (APTT) at 32.3 s, and thrombin time (TT) at 13.9 s. Notably, both the fibrinogen activity and antigen concentrations were reduced, with levels dropping to 0.91 and 1.24 g/L, respectively.

### 3.2 DNA Sequencing

**FIGURE 1:**
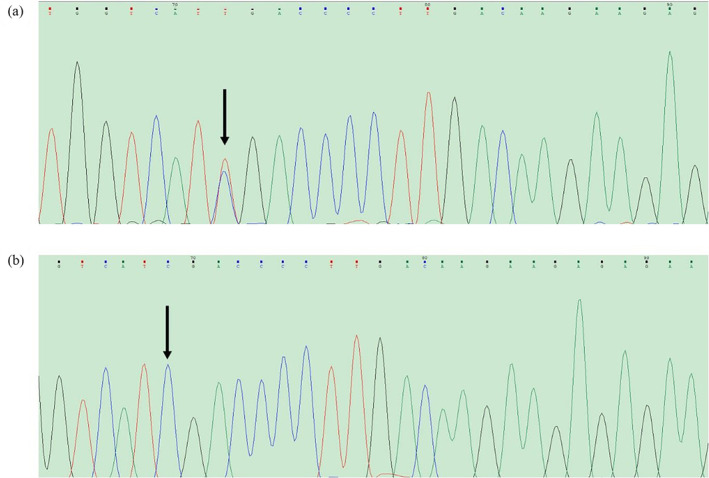
DNA sequence of exon 2 of FGB. (a) Point mutation C → T in the fibrinogen Bβ chain of proband; (b) Normal control point.

DNA sequencing analysis identified a heterozygous c.139C>T mutation (p.Arg17Stop) in exon 2 of the fibrinogen FGB gene (NM_005141), as shown in Figure 1. Importantly, no mutations were detected in the FGA and FGG genes.

### 3.3 Effect of the Mutation on Fibrinogen Synthesis and Secretion

**FIGURE 2:**
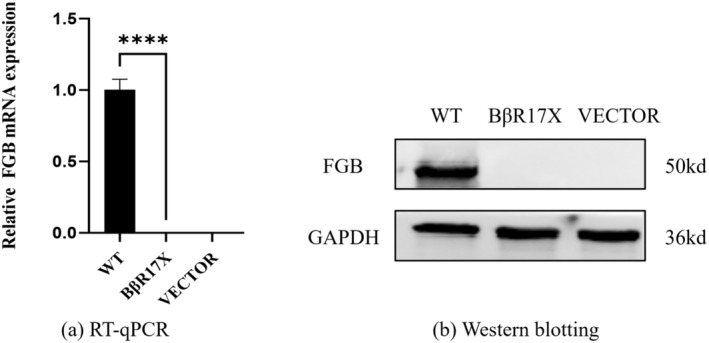
(a) RT‐qPCR results of vector, wild‐type, and mutant CHO cells after transfection; (b) Western blotting analysis of vector, wild‐type, and mutant CHO cells after transfection. ****, *p* < 0.0001.

**FIGURE 3:**
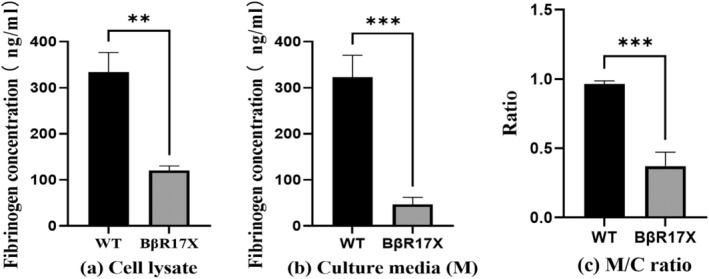
(a) The fibrinogen concentration of cell lysates. (b) The fibrinogen concentration of culture media and cell lysates. (c) The ratio of fibrinogen concentration in culture media to that in cell lysates. The fibrinogen concentrations were detected by ELISA. **, *p* < 0.01; ***, *p* < 0.001.

Expression of *FGB* mRNA and protein was absent in mutant cells, in contrast to wild‐type cells (Figure 2). Fibrinogen concentration in the supernatant was 322.9 ± 47.71 ng/mL for wild‐type cells and 46.82 ± 15.39 ng/mL for mutant cells (Figure 3a). Fibrinogen concentrations in the cell lysate were 334 ± 42.44 ng/mL for wild‐type cells and 120.4 ± 9.691 ng/mL for mutant cells (Figure 3b). The ratio of fibrinogen concentration between the cell supernatant and lysate was 0.96 ± 0.02 for wild‐type cells and 0.37 ± 0.1 for mutant cells (Figure 3c).

### 3.4 Conservation Analysis and Bioinformatics Analysis

**FIGURE 4:**
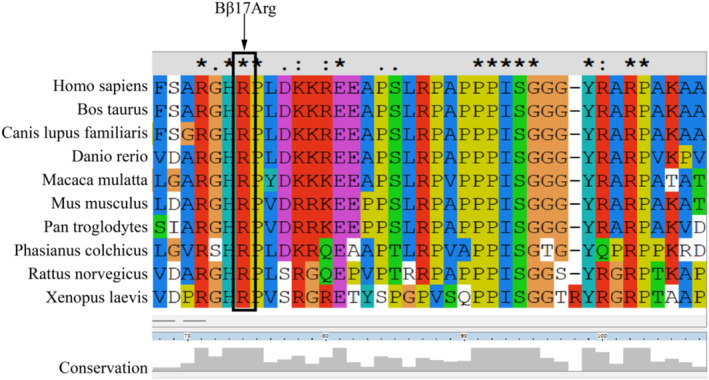
Multiple sequence alignment of the mutation site. '*', A fully conserved column, meaning that the residues in this column are identical; ':', The residues in this column have roughly similar molecular size and the same hydrophobicity or hydrophilicity, indicating that the residues are either identical or similar; '.', In the course of evolution, the molecular size and hydrophobicity/hydrophilicity of residues have been somewhat retained, but substitutions have occurred among dissimilar residues; ' ', A completely non‐conserved column.

The conservation analysis of ten species using ClustalX2.1 software demonstrated that the Bβ17Arg site is highly conserved (Figure 4). This indicates that the Bβ17Arg site is crucial for both the structure and function of the wild‐type FGB protein. The variant Bβ (p.Arg17Stop) was predicted to be “disease‐causing” by MutationTaster (http://mutationtaster.org/). It was also predicted to be “deleterious” by PROVEAN (http://provean.jcvi.org/). According to the American College of Medical Genetics and Genomics standards and guidelines (ACMG), the variant Bβ (p.Arg17Stop) can be classified as “Pathogenic” (PVS1 + PM2 + PP3 + PS3 + PS4).

### 3.5 Molecular Modeling of the Amino Acid Mutations

**FIGURE 5:**
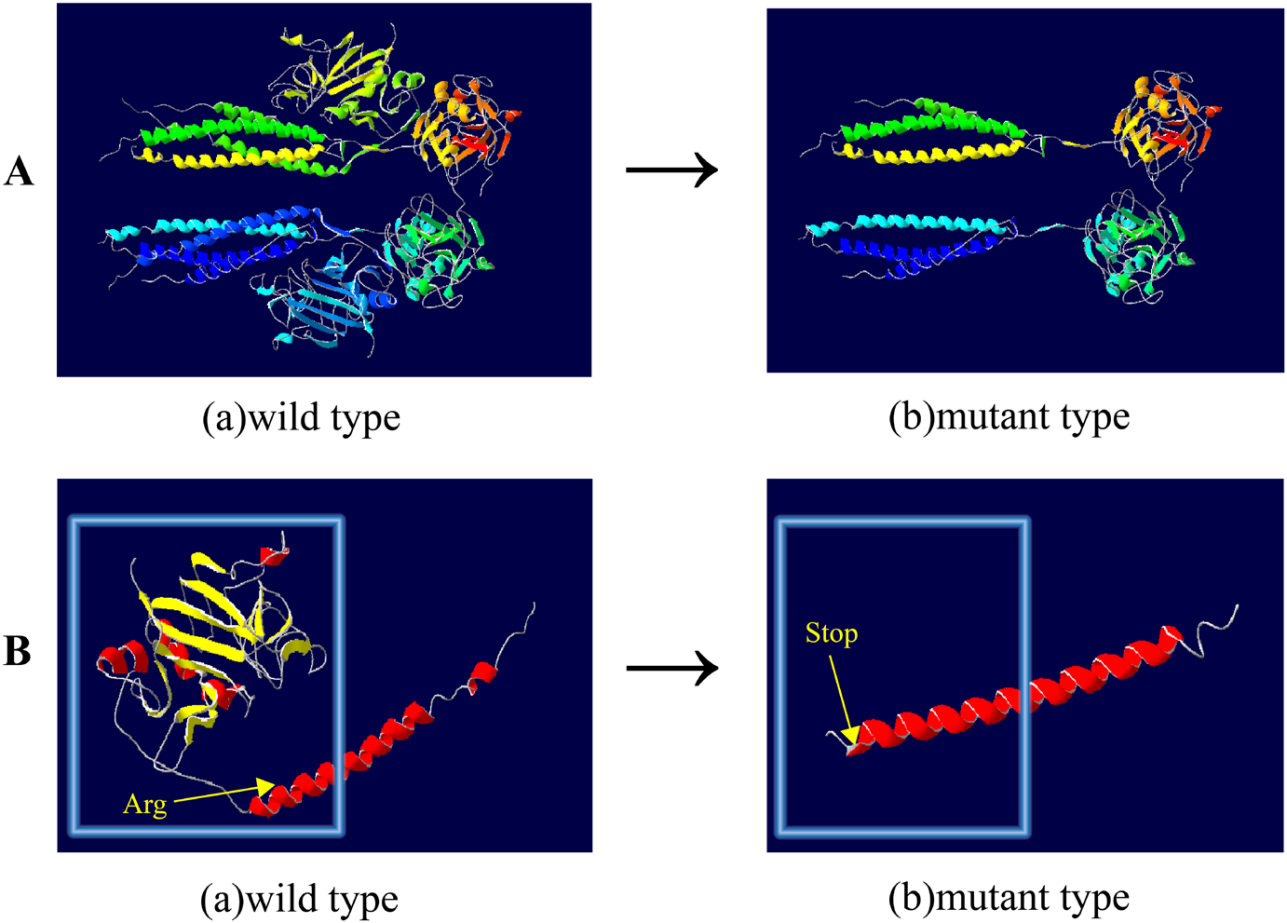
(A) Ribbon structure of the fibrinogen molecule: (a) wild‐type; (b) mutant type. (B) Ribbon structure of the fibrinogen Bβchain molecule: (a) wild‐type; (b) mutant type.

The Bβ17Arg residue is situated within the polymerization domain of the fibrinogen beta chain, a crucial region responsible for binding to the distal domain of another fibrin molecule (as depicted in Figure 5B). When this arginine (Arg) is replaced by a stop codon, translation of the subsequent amino acids is halted, leading to the production of a truncated Bβ‐chain that is 445 amino acids shorter than the normal Bβ‐chain. This truncation is predicted to cause a significant loss of secondary structural elements, including β strands, α helices, and turns, as well as the disruption of several hydrogen bonds (Figure 5A).

## 4 Discussion

**FIGURE 6:**
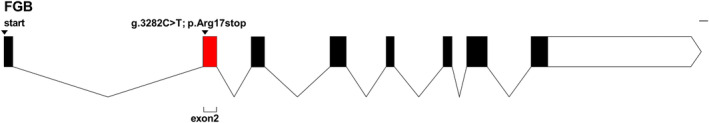
Schematic representation of the FGB genomic region spanning from exon 1 to exon 6. Exons are shown as boxes, whereas introns are represented by lines. Arrows indicate the positions of the BβR17× mutation.

In the coagulation function tests of the proband, fibrinogen concentrations measured by the Clauss method and the PT algorithm were decreased, registering 0.91 and 1.24 g/L, respectively. Subsequent analysis of gene sequencing results revealed a c.139C>T heterozygous mutation (p.Arg17Stop) in exon 2 of the fibrinogen *FGB* gene. The *FGB* genomic region spanning from exon 1 to exon 6 is shown in Figure 6. This mutation causes severe truncation of the fibrinogen Bβ chain. We compared the amino acid sequences of Bβ chains across 10 species using multiple sequence alignment, demonstrating that Bβp.P17 is highly conserved across these species. We analyzed the impact of the Bβ (p.Arg17Stop) mutation on the spatial structure of fibrinogen using homology modeling techniques. Analysis revealed that the fibrinogen C‐terminal domain was lost in the mutant, and the structure of the beta chain polymerization domain was altered. The loss of the fibrinogen C‐terminal domain seriously affects fibrinogen hexamer secretion [12]. Changes in the beta chain polymerization domain cause the mutant fibrin to fail in binding the distal domain of another fibrin, ultimately impairing fibrin polymerization and blood clot formation [13]. We utilized the bioinformatics tool MutationTaster (https://mutationtaster.org/) to predict the potential impact of the Bβ (p.Arg17Stop) mutation on protein structure and function. This mutation is predicted to be disease‐causing and may result in nonsense‐mediated decay (NMD). The NMD pathway acts as a surveillance mechanism, identifying aberrant mRNAs with premature termination codons (PTCs) due to nonsense variants or aberrant splicing events [14]. It efficiently removes these abnormal transcripts, preventing harmful protein synthesis and ensuring cellular integrity [14].

We identified eight patients carrying the *FGB* p.Arg17Stop mutation in the Human Fibrinogen Database (GEHT.org [25]) (Table 1). However, the genetic status of these patients is either homozygous or compound heterozygous, and there are no reports on the pathogenic mechanisms of the fibrinogen Bβ (p.Arg17Stop) mutation. We reported the first case of a *FGB* p.Arg17Stop mutation with a heterozygous gene status, and conducted research on the pathogenic mechanisms related to the fibrinogen Bβ (p.Arg17Stop) mutation. Through literature review, we discovered that patients with the homozygous p.Arg17Stop mutation were diagnosed with afibrinogenemia and exhibited varying degrees of bleeding symptoms; however, in this case, the patient with the heterozygous p.Arg17Stop mutation was diagnosed with hypofibrinogenemia and exhibited no clinical manifestations but may be at increased risk of hemorrhage during stressful situations such as trauma, surgery, or childbirth.

**TABLE 1:** Information of patients with Bβ(17)Arg > Stop mutation.

Biological phenotype	Clinical phenotype residence	Gender	Age at diagnosis	Gene status	Authors
Afibrinogenemia	Umbilical cord bleeding, gastrointestinal bleeding, and other relatively mild hemorrhagic symptoms (epistaxis, mouth bleeding, and menorrhagia)	Female	17	Homozyg	Asselta R et al. 2002 [23]
Afibrinogenemia	Bilateral cephalohematomas, asymptomatic thrombosis of the upper venous system	Male	1	Compound	Vu D et al. 2003 [18]
Hypofibrinogenemia	Six spontaneous abortions, Acute arterial thrombosis	Female	26	Compound	Asselta R et al. 2004 [19]/Castaman et al. 2009 [22]
Afibrinogenemia	N/A	N/A	N/A	Compound	Czwalinna A 2004 [25]
Afibrinogenemia	Umbilical cord bleeding, Bleeding persists after trauma and Intracranial hemorrhage	Male	6	Compound	Fang Y et al. 2005 [24]
Afibrinogenemia	Umbilical cord bleeding, gum bleeding, bruises	Female	4	Homozyg	Asselta R et al. 2015 [21]
Afibrinogenemia	Umbilical cord bleeding, gum bleeding, epistaxis	Female	6	Homozyg	Asselta R et al. 2015 [21]
Afibrinogenemia	Umbilical stump bleeding, recurrent soft tissue bleeds	Male	4	Compound	Young GA et al. 2018 [20]

To investigate the potential pathogenic mechanisms of this mutation, we constructed a plasmid in vitro, transfected it into CHO cells, and analyzed the mutation's effects on the *FGB* gene's transcription and translation, and on fibrinogen synthesis and secretion. As shown in Figure 2, the transfected mutant cells exhibited no mRNA and protein expression of the *FGB* gene, suggesting that the mutation impairs both the transcription and translation processes of the *FGB* gene. Three potential mechanisms might explain this outcome: In line with Mutation Taster's predictions, it is hypothesized that the NMD pathway eliminated the aberrant mRNAs, leading to the absence of *FGB* mRNA and protein expression in mutant cells [15]. This mutation could compromise mRNA stability, increasing its susceptibility to degradation or diminishing its translational efficiency [16]. Nonsense mutations yield proteins that are too short to fold properly or execute their biological functions, thus reducing protein expression levels [17]. We subsequently employed ELISA to quantify fibrinogen concentrations in the cell lysates and supernatants. Results showed that, although fibrinogen was present in the lysates and supernatants of mutant cells, levels were significantly lower than those in wild‐type cells. This suggests that the p.Arg17Stop mutation severely affects fibrinogen synthesis and secretion. It also indicates that, in the absence of β‐chain expression, it is still possible for the organism to synthesize some incomplete fibrinogen structures containing the α and γ chains, albeit with potentially limited functionality.

We report a case of hypofibrinogenemia with a heterozygous fibrinogen Bβ chain (p.Arg17Stop) mutation. This mutation significantly impairs both the synthesis and secretion of fibrinogen. Exploring the pathogenic mechanism of the fibrinogen Bβ chain (p.Arg17Stop) mutation will help clarify the structure–function relationship in the fibrinogen molecule and will enrich the genetic data on fibrinogen deficiencies.

## Author Contributions

Chen Qian: Conceptualization, data curation, formal Analysis, investigation, methodology, validation, visualization, writing – original draft, writing – review and editing. Chaoyu Huang: Investigation, data curation, visualization. Qinghui Luo: Investigation, data curation. Kaili Qin: Formal analysis, visualization. Lin Liao: Data curation. Qian Zhang: Visualization. Liqun Xiang and Jie Yan: Conceptualization, funding acquisition, methodology, resources, supervision, writing – review and editing. All authors have approved the manuscript and the submission.

## Ethics Statement

This study was reviewed and approved by the Medical Ethics Committee of the First Affiliated Hospital of Guangxi Medical University. Written informed consent was obtained from the individual for the publication of any potentially identifiable images or data included in this article. Written informed consent was obtained from the patient for the publication of this case report.

## Conflicts of Interest

The authors declare no conflicts of interest.

## Supporting information


**Table S1.** Reaction system of Real‐time PCR.

**Table S2.** Reaction conditions of Real‐time PCR.

**Table S3.** Primer sequence information.

## Data Availability

The data that support the findings of this study are available from the corresponding author upon reasonable request.
