# Supplementary material for: The β‐Chain Mutation p.Arg17Stop Impairs Fibrinogen Synthesis and Secretion: A Nonsense Mutation Associated With Hypofibrinogenemia
Source: J Clin Lab Anal. 2024 Dec 12;38(24):e25123. doi: 10.1002/jcla.25123 (PMC11659728; doi:10.1002/jcla.25123)
Supplement: Supplementary file 1 — Table S1. Reaction system of Real‐time PCR. [file JCLA-38-e25123-s003.docx]

The reaction system of Real Time PCR

| Name | volume |
| --- | --- |
| TB Green Premix Ex Taq II（Tli RNaseH Plus）（2X） | 10 μl |
| PCR Forward Primer（10 μM） | 0.8 μl |
| PCR Reverse Primer（10 μM） | 0.8 μl |
| ROX Reference Dye II（50X） | 0.4 μl |
| cDNA | 2 μl |
| RNase-Free water | 6 μl |
| Total | 20 μl |
