# Supplementary material for: The β‐Chain Mutation p.Arg17Stop Impairs Fibrinogen Synthesis and Secretion: A Nonsense Mutation Associated With Hypofibrinogenemia
Source: J Clin Lab Anal. 2024 Dec 12;38(24):e25123. doi: 10.1002/jcla.25123 (PMC11659728; doi:10.1002/jcla.25123)
Supplement: Supplementary file 2 — Table S2. Reaction conditions of Real‐time PCR. [file JCLA-38-e25123-s002.docx]

| Stage | reaction conditions |
| --- | --- |
| pre-denaturation(1 cycle) | at 95℃ for 30 s |
| PCR reaction(40 cycles) | denaturation at 95℃ for 5 s and annealing at 60℃ for 34s |

The reaction conditions of Real Time PCR
