# Supplementary material for: The β‐Chain Mutation p.Arg17Stop Impairs Fibrinogen Synthesis and Secretion: A Nonsense Mutation Associated With Hypofibrinogenemia
Source: J Clin Lab Anal. 2024 Dec 12;38(24):e25123. doi: 10.1002/jcla.25123 (PMC11659728; doi:10.1002/jcla.25123)
Supplement: Supplementary file 3 — Table S3. Primer sequence information. [file JCLA-38-e25123-s001.docx]

Primer sequence information

| Name | Sequences(5′→3′) |
| --- | --- |
| FGB-F | ACCTTCGTGTGCTTCGTTCA |
| FGB-R | CTGAATCACTGTCCATCCTCCA |
| β-actin-F | GTGCTATGTTGCTCTAGACTTCG |
| β-actin-R | ATGCCACAGGATTCCATACC |
